# Multi-Channel Singular Spectrum Analysis on Geocenter Motion and Its Precise Prediction

**DOI:** 10.3390/s21041403

**Published:** 2021-02-17

**Authors:** Xin Jin, Xin Liu, Jinyun Guo, Yi Shen

**Affiliations:** 1 College of Geodesy and Geomatics, Shandong University of Science and Technology, Qingdao 266590, China; skdjinxin1010@sdust.edu.cn (X.J.); guojy@sdust.edu.cn (J.G.); 2 School of Geographic Sciences, Xinyang Normal University, Xinyang 464000, China; shenyi@xynu.edu.cn

**Keywords:** multi-channel singular spectrum analysis, geocenter motion, prediction, autoregressive moving average

## Abstract

Geocenter is the center of the mass of the Earth system including the solid Earth, ocean, and atmosphere. The time-varying characteristics of geocenter motion (GCM) reflect the redistribution of the Earth’s mass and the interaction between solid Earth and mass loading. Multi-channel singular spectrum analysis (MSSA) was introduced to analyze the GCM products determined from satellite laser ranging data released by the Center for Space Research through January 1993 to February 2017 for extracting the periods and the long-term trend of GCM. The results show that the GCM has obvious seasonal characteristics of the annual, semiannual, quasi-0.6-year, and quasi-1.5-year in the X, Y, and Z directions, the annual characteristics make great domination, and its amplitudes are 1.7, 2.8, and 4.4 mm, respectively. It also shows long-period terms of 6.09 years as well as the non-linear trends of 0.05, 0.04, and –0.10 mm/yr in the three directions, respectively. To obtain real-time GCM parameters, the MSSA method combining a linear model (LM) and autoregressive moving average model (ARMA) was applied to predict GCM for 2 years into the future. The precision of predictions made using the proposed model was evaluated by the root mean squared error (RMSE). The results show that the proposed method can effectively predict GCM parameters, and the prediction precision in the three directions is 1.53, 1.08, and 3.46 mm, respectively.

## 1. Introduction

The center of mass (CM) of the Earth is defined as the center of the mass of the entire Earth including the solid Earth, ocean, and atmosphere, which is consistent with the International Terrestrial Reference Frame (ITRF) and International Astronomical Union (IAU) resolutions [1,2,3]. Sea level change, glacier melting, crustal rebound, atmospheric circulation, and mantle convection result in the movement of CM relative to the center of the figure (CF) of the Earth surface, which reflects the global mass redistribution and the interaction between solid Earth and mass loading [4,5]. The geocenter motion (GCM) is the basis for constructing and maintaining ITRF reference frame [6,7,8,9]. The GCM has an important impact on the reference frame transformation of Satellite Laser Ranging (SLR), Global Navigation Satellite System (GNSS), and Doppler Orbitography and Radio-positioning Integrated by Satellite (DORIS) system [10,11]. It is also an important topic for studying the Earth’s mass redistribution, such as ocean tide, glacial isostatic adjustment, atmospheric and ocean circulation, and geodynamic process in the Earth’s interior [12,13,14].

GCM can be obtained by solving spatial geodetic data. For example, SLR data were processed to estimate effectively GCM [15,16]. GNSS data were also used to determine GCM [17]. Although there are more international GNSS service stations in the world, the precision of GNSS-derived GCM due to the GNSS satellites’ elevation angles and serious collinearity errors is lower than that of SLR-derived products [18,19]. The DORIS-derived GCM in the Z direction is very different from the other two directions, and its precision is only up to centimeter level. DORIS-derived GCM has the lowest precision among the series estimated by these three space geodetic techniques [20,21]. The time series of GCM in different time-span can be effectively estimated by using the SLR data of Lageos-1, Lageos-2, or other geodynamical satellites. For example, Center for Space Research (CSR)’s GCM products, showing the most reliable sensitivity to CM, are adopted to determine ITRF origin [22,23].

The wavelet transformation, least-squares spectral analysis, and singular spectrum analysis (SSA) have been used to discover the characteristics of GCM in X, Y, and Z directions [10,24]. However, when analyzing the GCM, these methods cannot take into account the correlation of GCM in all directions. Multi-channel singular spectrum analysis (MSSA), as the extended form of SSA, is one of the effective statistical data analysis methods in oceanography, geoscience, meteorology, and other fields [25,26,27,28]. The MSSA is a method for analyzing non-linear time series. It is able to denoise data, extract periodic oscillation signals, and identify trends from multidimensional time series, and it can build prediction models as well [29,30]. Compared with SSA, during the process of multidimensional time series, the correlations among different channels are taken into account, so we apply the MSSA method to analyze GCM for better extracting periodic signals of GCM.

The monitoring and modeling of GCM is a key issue for constructing a millimeter-level, dynamic, and real-time global reference frame. However, due to the complexity of obtaining multi-source observations and data processing, the GCM parameters cannot be obtained in real time or quasi-real-time [9,31]. Therefore, introducing the MSSA model into GCM prediction, this paper proposes a GCM prediction method combing the linear model (LM), MSSA, and autoregressive moving average model (ARMA).

SLR-derived GCM series from January 1993 to February 2017 updated by the CSR, the Texas University at Austin, were used to study the GCM variations in this paper. The trend and periodic variations of GCM are investigated by using MSSA. Finally, based on historical GCM data, the method combing LM, MSSA, and ARMA models was used to predict GCM parameters.

## 2. Materials and Methods

### 2.1. SLR-Derived GCM Products

The GCM products used in this paper are obtained from CSR at University of Texas website [32]. The GCM monthly solution products (GCN_L1_L2_30d_CF-CM) were solved by University of Texas Orbit Determination Program (UTOPIA) and Large Linear System Solver (LLISS) from SLR data of geodynamical satellites (e.g., Lageos-1/2) in SLRF2014 [33,34,35]. In data processing, models such as planetary ephemeris, earth’s gravity field model (GGMO5C), and ocean tide model (GOT00.2) were used to correct the influence of perturbation forces, such as N-body perturbation, tidal perturbation, and relativistic effects [6,23,36]. CF-CM is intended to reflect the true degree-1 mass variations without being affected by the higher-degree site loading effects (particularly at the annual frequency) [35]. The GCM products are often used to study the local and global mass balance with Gravity Recovery and Climate Experiment (GRACE) and are currently the best geocenter coordinate result recognized internationally [23]. Here, we download the GCM products from January 1993 to February 2017.

### 2.2. Multi-Channel Singular Spectrum Analysis

There is a time series $x_{li}\left(l=1,\ldots ,L;i=1,\ldots ,N\right)$ in which *l* is channel number and *i* is time series number. The rank of $x_{li}$ is arranged according to the time delay phase space *M* (1≤M≤N/2) that is the window length and also called the step number of time lag. The integer multiple of the main periods is generally chosen as one window length in MSSA [30,37].

The trajectory matrix of the channel l is

(1) $$X_{l}=\left[\begin{array}{cccc} x_{l1} & x_{l2} & \cdots & x_{lK}\\ x_{l2} & x_{l3} & \cdots & x_{lK+1}\\ \vdots & \vdots & \vdots & \vdots \\ x_{lM} & x_{lM+1} & \cdots & x_{lN} \end{array}\right]$$

where K=N−M+1. The multi-channel trajectory matrix can be indicated as

(2) $$X=[X_{1},X_{2},\cdots ,X_{L}{]}^{T}$$

Matrix X has L×M rows and N−M+1 columns. Similar to SSA, the next step is to decompose the singular value of X. We define the matrix $S=XX^{T}$, where $X^{T}$ is the transposed matrix of X. Suppose that $\lambda_{1},\cdots ,\lambda_{M}$ are the eigenvalues of matrix **S**, that is, the singular values. These eigenvalues are arranged in the descending order. The larger singular value generally represents the larger energy signal, and the smaller one corresponds to the noise part. Matrix **X** can be expressed in the elementary matrix as

(3) $$X=P_{1}+P_{2}+\cdots +P_{D}$$

where *D* represents the number of singular values, and $P_{i}=S_{i}U_{i}V_{i}^{T}$ in which $U_{i}$ is the temporal empirical orthogonal function and $V_{i}$ is the temporal principal components.

The GCM time series contain different signals, such as annual term and semi-annual term. It is necessary to use the w-correlation method [38] to merge elementary matrix $P_{i}$ representing the same signal into a group. Suppose that the time series after reconstruction of each elementary matrix $P_{i}$ is $Y_{i}$; then, the correlation of any two reconstructed time series can be expressed by w-correlation as

(4) $$\rho_{i,j}^{w}=\frac{\left(Y^{\left(i\right)},Y^{\left(j\right)}\right)}{{\left\|Y^{i}\right\|}_{w}{\left\|Y^{j}\right\|}_{w}},(1\leq i,j\leq N)$$

where ${\left\|Y^{i}\right\|}_{w}=\sqrt{\left(Y^{\left(i\right)},Y^{\left(i\right)}\right)}$, $(Y^{\left(i\right)},Y^{\left(j\right)})=\sum_{k=1}^{N}w_{k}y_{k}^{i}y_{k}^{j}$, and $w_{k}=\min(k,M,N-k)$. The larger the absolute value of $\rho_{i,j}^{w}$ is, the greater the correlation of the corresponding components of *i* and *j*, which should be classified as the same periodic signal component. Then, the corresponding trajectory matrix has been built.

The corresponding group of trajectory matrix is converted into a new time series with the length of *N*, which is called the reconstructed component [39]. Then, the reconstructed component (RC) is

(5) $$x_{li}^{k}=\left\{\begin{array}{ll} \frac{1}{i}\sum_{j=1}^{i}a_{i-j}^{k}E_{lj}^{k} & 1\leq i\leq M-1\\ \frac{1}{M}\sum_{j=1}^{M}a_{i-j}^{k}E_{lj}^{k} & M\leq i\leq N-M+1\\ \frac{1}{N-i+1}\sum_{j=i-N+M}^{M}a_{i-j}^{k}E_{lj}^{k} & N-M+2<i\leq N \end{array}\right.$$

## 3. Analysis of GCM

### 3.1. GCM Seasonal Variations

**Figure 1:**
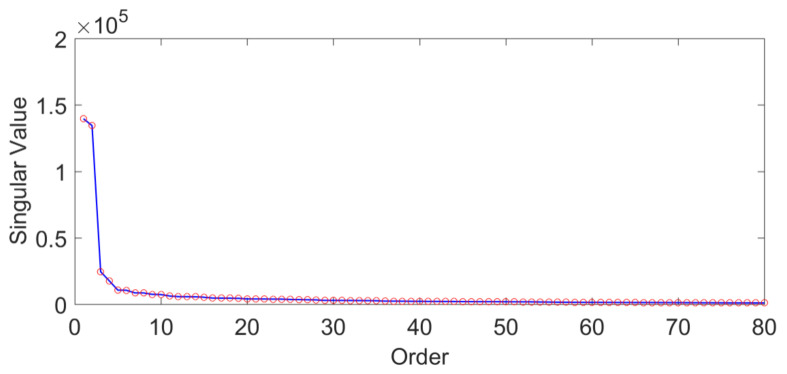
Singular values of GCM series determined by MSSA.

The GCM series comprising monthly data (L=290) from January 1993 to February 2017 are chosen to study GCM variations. The MSSA method is used to analyze the original time series without the linear trend to explore the time-varying law of GCM. The trajectory matrix of GCM is decomposed by selecting the window M=108 (month), which is determined by the period of the annual term of GCM and many practical experiments. Singular values in descending order are shown in Figure 1.

**Figure 2:**
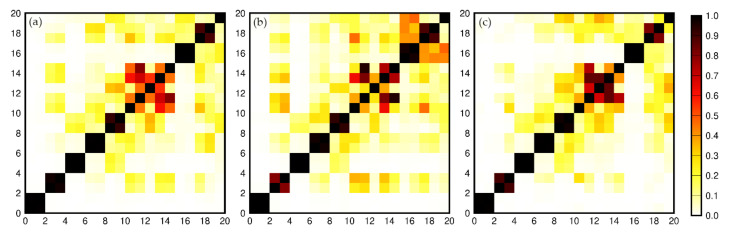
W-correlations for the first 20 reconstructions. (**a**) X direction; (**b**); Y direction;(**c**) Z direction.

As shown in Figure 1, the values starting from the 20th singular value are already smaller than the first four previous singular values, and the values after the 20th order change smoothly so that they can be ignored. The w-correlations $\rho_{i,j}^{w}$ of the first 20 reconstruction parts of GCM in the three directions except for the trend are shown in Figure 2.

**Figure 3:**
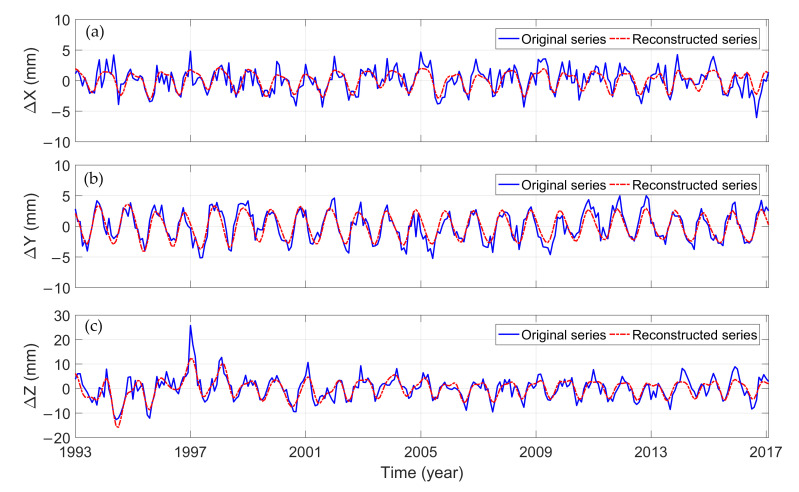
Time series of GCM from January 1993 to February 2017. (**a**) X direction; (**b**) Y direction, and (**c**) Z direction.

The greater w-correlation $\rho_{i,j}^{w}$ means the corresponding components belong to the same periodic term. As shown in Figure 2, the data in the time series in the X direction are not completely separated from each other when i>10, and the separation effect in both Y and Z directions also deteriorates after i>10, which may be caused by noise. Hence, the first 10 groups (RC1, RC2, …, and RC10) are used to reconstruct the GCM series. RC1 and RC2 represent a periodic term in the original series; RC3 and RC4 represent the other one; RC5 and RC6, RC7 and RC8, and RC9 and RC10 can be combined into one periodic component, respectively. Figure 3 shows the original GCM series in X, Y, and Z directions without the linear trend, and the time series reconstructed by the first 10-order reconstruction components.

As shown in Figure 3, the fluctuation ranges of raw GCM in the X and Y directions are smaller than that in the Z direction. Although the offset of CM relative to CF in the Z direction is large, the fluctuation amplitude is small, and most of them are negative.

The correlation coefficients between the time series reconstructed by the first 10 RCs and the corresponding original time series in the X, Y, and Z directions are 0.73, 0.86, and 0.83, respectively, which indicates that they have good consistency. It shows that the MSSA can effectively extract relatively complete information about the main components in the three directions.

**Figure 4:**
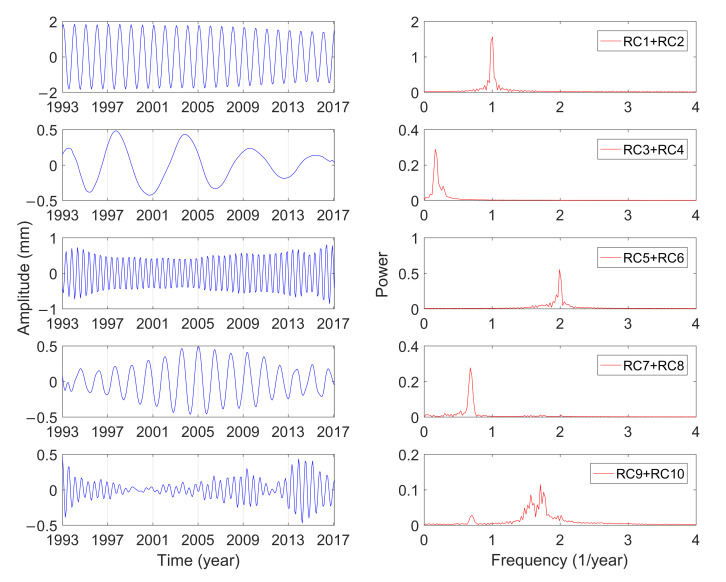
Combination of reconstructed component (RC) and the power spectrum analysis in the X direction.

**Figure 5:**
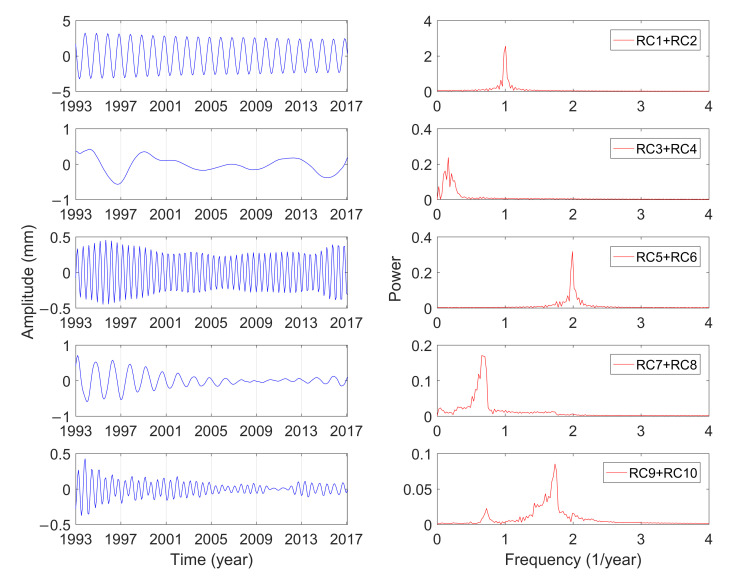
Combination of RC and the power spectrum analysis in the Y direction.

**Figure 6:**
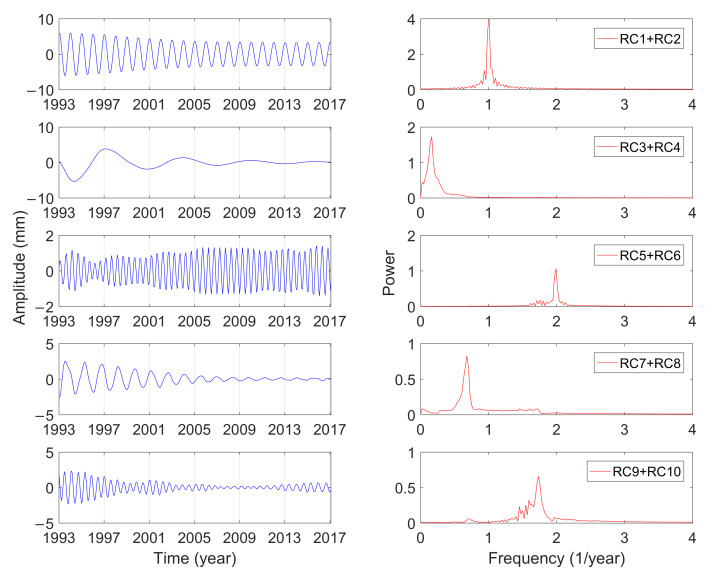
Combination of RC and the power spectrum analysis in the Z direction.

Table 1 shows the singular spectrum values and the variance contributions of the GCM series calculated by MSSA from January 1993 to February 2017. The variance contribution of the first 10-order reconstruction components has reached 65.79%, which can characterize the variations of GCM effectively. Furthermore, the variance contribution of RC1 and RC2 is significantly larger than that of other components, which indicates that the corresponding cyclophysis is most obvious. According to the principle of w-correlation, the first 10-order reconstruction components can be combined into five periodic terms, which were analyzed by using the fast Fourier transform (FFT) [40]. Figure 4, Figure 5 and Figure 6 show the reconstructed components and the corresponding power spectrum in the three directions.

As shown in Figure 4, Figure 5 and Figure 6, the five main periodic terms of GCM in three directions are basically the same. There is the annual term, semi-annual term, quasi-0.6-year term, quasi-1.5-year term, and long-term term. The periods are respectively 0.99 years, 0.5 years, 0.58 years, 1.47 years, and 6.09 years. The variance contributions of these five main periodic components in the three directions are different, and annual characteristics make great domination. Table 2 shows the amplitudes of the three-direction offset of the annual GCM estimated by different scholars. The idea of using SLR data to estimate GCM is dominant and has been widely proven to be reliable in the past ten years. Excluding the results of this paper, the estimated average amplitudes of the three-direction X, Y, and Z of the annual term in Table 2 are 2.4, 2.9, and 4.9 mm. For the Y and Z directions, the estimated results in this paper are more consistent with the average value (2.8 and 4.4 mm), and the amplitude in the X direction is closer to the result calculated by GPS loading/OBP/GRACE.

The annual periodic oscillations in the three directions are relatively stable, and the periodicity is the most obvious. The valley value of the annual variations of GCM in the X direction occurs from August to September, and the peak value appears from February to March. The valley value of the annual variations of GCM in the Y direction occurs from May to June, and the peak value appears from November to December. The valley value of the annual variations of GCM in the Z direction occurs from July to August, and the peak value appears from January to February.

The amplitude of the semi-annual term changes with time, which is more in line with the actual variations of GCM. The traditional least-squares spectrum analysis can only give a constant amplitude of the period term. Although the semi-annual variations are shown in these three directions, the corresponding amplitude variations characteristics are not the same. The semiannual amplitudes in the three directions vary from 0.3 to 0.8 mm, 0.2 to 0.5 mm, and 0.4 to 1.5 mm, respectively, and they have been slowly increasing in the last 20 years. The analysis is consistent with the results of Zhang et al. [44], whose amplitude changes in three directions are within 0.5 mm, 0.1 to 0.6 mm, and 0.3 to 1.5 mm, respectively. The cyclophysis of quasi-1.5-year and quasi-0.6-year, one of the five main periods of GCM, also exhibits strong seasonal characteristics. The oscillation characteristics of quasi-1.5-year and quasi-0.6-year period in the Y and Z direction are similar, and the amplitude gradually decreases with time. The oscillation of the quasi-1.5-year period in the X direction has obvious fluctuations and reached its maximum in February 2005. The seasonal character of the quasi-0.6-year period in the X direction is obvious, which may be due to the inclusion of many other signals in the period.

The annual term, semi-annual term, quasi-0.6-year term, and quasi-1.5-year term mentioned above belong to the seasonal period of GCM. The solar radiation, changes in the gravitational field, and other Earth external energy cause the surface mass redistribution of land water, ocean, and atmosphere, which results in the significant seasonal GCM. The major reason for the seasonal cycle of GCM is the seasonal variations of land water storage [45,46]. The exchange of water mass in the Earth’s hemisphere has a clear annual cycle. Greater water mass in the northern hemisphere appears during June–August, while it appears during December–February in the southern hemisphere [47,48]. The peak and valley values of the annual term in the Z direction may be the reflection of water mass redistribution.

The long period terms in the X, Y, and Z directions analyzed by MSSA are all 6.09 years. The major reason for the secular variations of the center of mass of the Earth system is the glacial isostatic adjustment. The influence of the glacial ablation on the solid Earth causes a GCM velocity of less than 1 mm/yr [49]. The amplitude of the long-period term in the X direction is maintained within 0.5 mm, but the amplitude in the Y and Z directions has a sudden increase of 1 mm in 1997, which may be caused by the El Nińo that was strongest in the 20th century [24].

### 3.2. GCM Trend Variations

**Figure 7:**
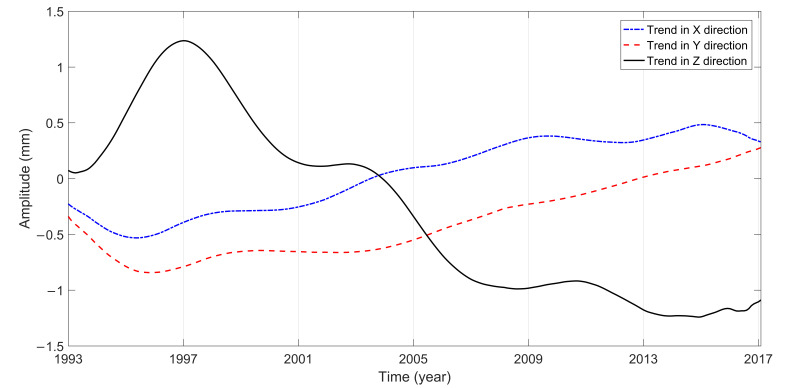
GCM trend variations by using MSSA.

The GCM series in the X, Y, and Z directions are directly decomposed and analyzed by using MSSA to determine their trends. The GCM trend variations from January 1993 to February 2017 are shown in Figure 7. The secular velocity of GCM is calculated by the least-square method. Table 3 shows the comparisons among the proposed method and the reported methods.

As shown in Figure 7, the trend variations in the three directions of GCM are nonlinear. After January 1997, the moving direction of GCM in the three directions remained stable. It can be seen from Table 3 that the long-term speed of GCM obtained from different data and time has a relatively large difference, and there is no accurate reference standard at present. In this paper, the variation rates in X, Y, and Z are 0.05, 0.04, and −0.10 mm/yr, respectively, all of which are less than 1 mm/yr. After 1997, the long-term variation rate of GCM in the Z direction is negative, which may be caused by glacial isostatic adjustment [50].

Due to the local expansion of the zero-degree hemisphere and the compression of the 180-degree hemisphere, the variation rate of GCM in the X direction is positive, which shows that CM moves toward the X direction relative to CF. The maximum variation of atmospheric pressure occurs in Central Asia [47,51]. The solid Earth is a viscous elastomer so that the maximum fluctuation of atmospheric pressure in Central Asia may have a certain impact on GCM in the Y direction, which results in the positive of the secular velocity.

## 4. Prediction of GCM

### 4.1. Principle of Prediction

**Figure 8:**
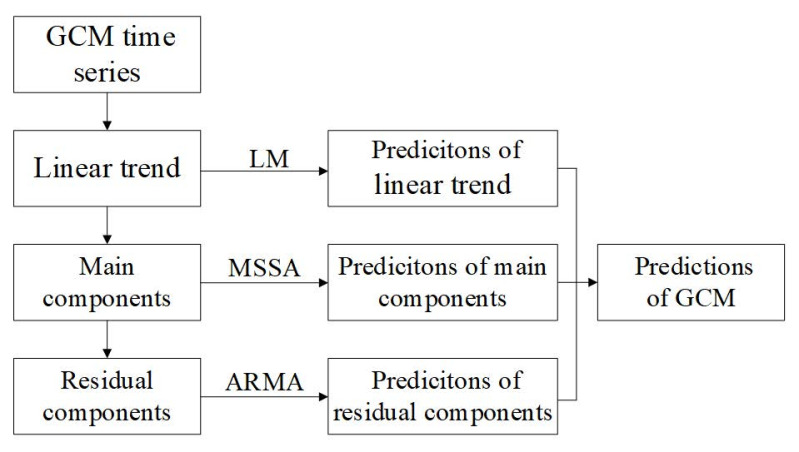
The prediction method of GCM.

With the continuous development and improvement of space technology, there is a growing demand for the prediction parameters of GCM, such as millimeter level, dynamic real-time global reference framework, and high-precision satellite navigation and positioning [8,55]. Therefore, to meet this demand, the method combing LM, MSSA, and ARMA is applied to predict the GCM parameters. The flow chart of prediction is shown in Figure 8.

(1) First, perform linear fitting on the GCM series, establish a linear model, and make predictions. Assuming that the GCM time series is $X_{t}$, the least squares method is used to linearly fit it, and the model is expressed as:
  (6) $$X_{t}=\beta_{0}+\beta_{1}t$$
  where $\beta_{0}$ is a constant, and $\beta_{1}$ represents the linear trend change rate.
(2) MSSA is used to decompose the GCM series of the de-linear trend, and the appropriate main component terms are selected for the main components prediction of the GCM. The prediction method for main components by MSSA is as follows:
  - The number of predictions of GCM is *N*; assuming that the time series of GCM without linear trend is $Y_{t}$, *N* zeros are added at the end of $Y_{t}$ to form a new prediction sequence $Y_{t+N}$;
  - The new predicted sequence $Y_{t+N}$ is decomposed by MSSA, and the *N* values at the end of the first RC (RC1) are used to replace the corresponding prediction values of the new sequence. This process is repeated until the RMS value of the two replacements data is less than 0.001 mas;
  - RC2 is added to reconstruct the prediction data; that is, the prediction data is obtained by linear superposition of RC1 and RC2. Step 2 is repeated until RC1...RCi is linearly added to the prediction data, and the predictions using MSSA can be obtained.
(3) ARMA is used to model and predict the residual components, which are the differences between the GCM series of the de-linear trend and reconstructed components by MSSA. Assuming that the series of residual items is $Z_{t}$, the ARMA model is expressed as:
  (7) $$Z_{t}=c+\sum_{i=1}^{p}\phi_{i}Z_{t-i}+\varepsilon_{t}+\sum_{j=1}^{q}\theta_{j}\varepsilon_{t-j}$$
  where *p* represents the order of the AR model, *q* represents the order of the MA model, *c* is the constant term of the ARMA model, $\phi_{i}$ is the autoregressive coefficient at time *t-i*, $\varepsilon_{t}$ is the error term at time *t*, and $\theta_{j}$ is the moving average coefficient at time *t-j*. The extended autocorrelation function (EACF) is selected to determine the order *p* and *q* of the AR model and the MA model through the maximum likelihood estimation method. The model parameters are estimated to build the ARMA model for predicting the residual items.

Finally, the GCM predictions are obtained by adding the predictions of the LM, MSSA, and ARMA models.

### 4.2. Results and Analysis

This paper selects the GCM series from January 1993 to February 2017 provided by CSR for prediction research. Taking the data from January 1993 to the starting time of forecast as the training data, seven 2-year forecast experiments were carried out. The starting time was January, May, and September 2013; January, May, and September 2014; and January 2015, respectively. Through the analysis in Section 3, the MSSA window and main components were selected as 108 and 10. Through EACF analysis, it is found that ARMA(0,6), ARMA(0,11), and ARMA(3,9) have good applicability to the residual items in the X, Y, and Z directions of GCM, respectively. The precision of predictions made using the proposed model was evaluated by the root mean squared error (RMSE).

**Figure 9:**
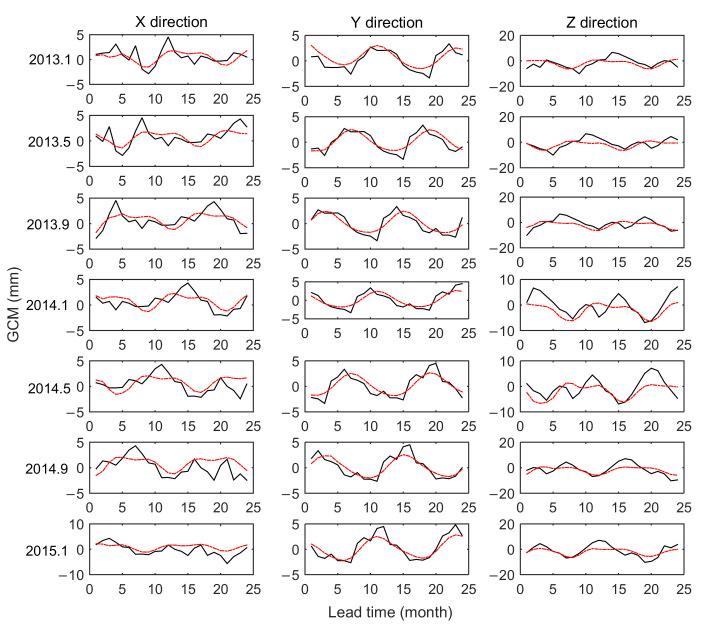
Comparison of GCM series (black line) with predictions of LM+SSA+MLP (red line).

Figure 9 shows the comparison between the predictions and the GCM series in the three directions. The black line in the figure represents the GCM series, and the red line represents the predicted values. The statistics of the prediction precision made using the LM+MSSA+ARMA model are shown in Table 4. 

As shown in Figure 9 and Table 4, the combined model has the best prediction precision when the prediction length is 6 months. The RMSE in the X, Y, and Z directions are 1.29 mm, 1.03 mm, and 3.29 mm, respectively, and the precision of the X and Y directions are all within 1.5 mm. The precision of the Z direction is relatively poor, which may be caused by the large change in the amplitude of GCM in the Z direction.

With the increase of the prediction length, the predicted precision shows a downward trend as a whole, reaching the maximum when the prediction length is 2 years, and the prediction precision in the three directions is 1.53 mm, 1.08 mm, and 3.46 mm, respectively. In general, the LM+MSSA+ARMA model has good performance in short-term, medium-term, and long-term predictions of GCM.

## 5. Conclusions

The variations of GCM can reflect the redistribution of the Earth’s mass. To study the time-varying characteristics of GCM in the X, Y and Z directions, MSSA was used to analyze the GCM series from January 1993 to February 2017 released by CSR. The analysis shows that the seasonal variations periods are 0.99, 0.5, 0.58, and 1.47 years, and the long period term is 6.09 years in the X, Y, and Z directions, which shows that the MSSA can well display and amplify the main periodic signals of the GCM series. The annual characteristics in the three directions are the most obvious and the wave oscillation is stable, with amplitudes of 1.7, 2.8, and 4.4 mm. The GCM in the X, Y, and Z directions are directly analyzed by using MSSA to determine its non-linear trends. The results show that the non-linear trends of the three directions are 0.05, 0.04, and −0.10 mm/yr, respectively. The migration velocity in the Z direction is obviously higher than that in the X and Y directions, which may be mainly caused by the water mass redistribution in the northern and southern hemispheres.

To obtain precision GCM prediction parameters, the method combining LM, MSSA, and ARMA models was used to predict GCM for 2 years into the future. The results show that the LM+MSSA+ARMA model can effectively predict GCM parameters, and the prediction precision in the three directions is 1.53, 1.08, and 3.46 mm, respectively.

## Figures and Tables

**Table 1:** Singular values and variance contributions of the first 10 orders based on MSSA.

Order/RC	Singular Spectrum Value	Variance Contribution	Cumulation
1	139,651.7	24.90%	24.90%
2	134,495.7	23.98%	48.88%
3	24,465	4.36%	53.24%
4	17,625.2	3.14%	56.38%
5	10,608.3	1.89%	58.27%
6	10,477.4	1.87%	60.14%
7	8517.8	1.52%	61.66%
8	8476.8	1.51%	63.17%
9	7423.4	1.32%	64.49%
10	7285.8	1.30%	65.79%

**Table 2:** Amplitude of GCM annual variations (mm).

Literature	Data	X	Y	Z
This paper	SLR(L1/L2)	1.7 ± 0.1	2.8 ± 0.1	4.4 ± 0.1
Altamimi et al. (2011) [9]	SLR(ILRS)	2.6 ± 0.1	3.1 ± 0.1	5.5 ± 0.3
Cheng et al. (2013) [41]	SLR(5 satellites)	2.7 ± 0.2	2.8 ± 0.2	5.2 ± 0.2
Ries et al. (2016) [35]	SLR(L1/L2)	2.8	2.5	5.8
Wu et al. (2010) [42]	GPS loading/OBP/ GRACE	1.8 ± 0.1	2.7 ± 0.1	4.2 ± 0.2
Wu et al. (2014) [43]	GPS loading/OBP/ GRACE	1.9	3.3	3.7

**Table 3:** Secular velocity of GCM in different studies (mm/yr).

Literature	Data	X	Y	Z	Time Span
This paper	SLR	0.05 ± 0.003	0.04 ± 0.004	−0.10 ± 0.01	1993–2017.2
Guo et al. (2009) [24]	SLR	−0.26 ± 0.02	0.43 ± 0.02	0.50 ± 0.02	1993–2006
Kuzin et al. (2010) [52]	DORIS/INA	−1.19 ± 0.07	−0.12 ± 0.07	−0.28 ± 0.31	1993–2009
Rietbroek et al. (2012) [53]	GRACE/Jason1/GIA	−0.28	0.43	−1.08	2003–2008
Sun et al. (2016) [54]	GRACE/OMCT/ICE-5G_VM2	−0.03 ± 0.03	0.11 ± 0.02	−0.21 ± 0.04	2002.6–2014.5
Sun et al. (2016) [54]	GRACE/OMCT/ICE-6G_VM5a	−0.06 ± 0.03	0.07 ± 0.02	−0.33 ± 0.04	2002.6–2014.5

**Table 4:** Statistical precision of linear model (LM)+MSSA+ARMA model (mm).

Lead Prediction	6 Month			12 Month			24 Month		
	X	Y	Z	X	Y	Z	X	Y	Z
Max	2.92	1.53	7.11	3.28	2.34	7.11	3.28	2.34	7.34
Min	−2.34	−2.19	−6.23	−2.34	−2.19	−6.60	−4.80	−2.19	−6.60
Mean	0.14	−0.15	0.58	0.08	−0.14	0.86	−0.32	−0.07	0.91
RMSE	1.29	1.03	3.29	1.35	1.08	3.45	1.53	1.08	3.46

## Data Availability

The data presented in this study are openly available in “http://ftp.csr.utexas.edu/pub/slr/geocenter/”.
